# Monocular Depth Estimation from a Fisheye Camera Based on Knowledge Distillation

**DOI:** 10.3390/s23249866

**Published:** 2023-12-16

**Authors:** Eunjin Son, Jiho Choi, Jimin Song, Yongsik Jin, Sang Jun Lee

**Affiliations:** 1 Division of Electronic Engineering, Jeonbuk National University, 567 Baekje-daero, Deokjin-gu, Jeonju 54896, Republic of Korea; eunjinson@jbnu.ac.kr (E.S.); jihochoi@jbnu.ac.kr (J.C.); jimin_song@jbnu.ac.kr (J.S.); 2 IT Convergence Research Section, Electronics and Telecommunications Research Institute (ETRI), Daegu 42995, Republic of Korea; yongsik@etri.re.kr

**Keywords:** monocular depth estimation, supervised depth estimation, knowledge distillation, fisheye camera, parking lot dataset

## Abstract

Monocular depth estimation is a task aimed at predicting pixel-level distances from a single RGB image. This task holds significance in various applications including autonomous driving and robotics. In particular, the recognition of surrounding environments is important to avoid collisions during autonomous parking. Fisheye cameras are adequate to acquire visual information from a wide field of view, reducing blind spots and preventing potential collisions. While there have been increasing demands for fisheye cameras in visual-recognition systems, existing research on depth estimation has primarily focused on pinhole camera images. Moreover, depth estimation from fisheye images poses additional challenges due to strong distortion and the lack of public datasets. In this work, we propose a novel underground parking lot dataset called JBNU-Depth360, which consists of fisheye camera images and their corresponding LiDAR projections. Our proposed dataset was composed of 4221 pairs of fisheye images and their corresponding LiDAR point clouds, which were obtained from six driving sequences. Furthermore, we employed a knowledge-distillation technique to improve the performance of the state-of-the-art depth-estimation models. The teacher–student learning framework allows the neural network to leverage the information in dense depth predictions and sparse LiDAR projections. Experiments were conducted on the KITTI-360 and JBNU-Depth360 datasets for analyzing the performance of existing depth-estimation models on fisheye camera images. By utilizing the self-distillation technique, the AbsRel and SILog error metrics were reduced by 1.81% and 1.55% on the JBNU-Depth360 dataset. The experimental results demonstrated that the self-distillation technique is beneficial to improve the performance of depth-estimation models.

## 1. Introduction

Artificial intelligence and computer vision techniques have been widely utilized in industry applications including intelligent vehicles and mobile robots. In these systems, low-cost and small-sized camera sensors offer many benefits for understanding the surrounding environments by providing spatially high-resolution information. Specifically, fisheye cameras have the advantage of covering wider areas compared to traditional rectilinear lens cameras. By intentionally generating strong distortion, fisheye cameras produce hemispherical images that cover more than 180° of the diagonal field of view (FOV). Due to the benefit of the wide FOV, there have been increasing demands for fisheye cameras in visual-recognition systems, which require complete coverage without any blind spots. Fisheye cameras have been utilized in implementing surveillance systems, self-driving vehicles, and autonomous parking systems.

Monocular depth estimation is a task that predicts pixel-level distances from a single RGB image. It is a fundamental building block of visual-recognition systems in intelligent robots, providing 3D information of the surrounding environments. By estimating distances to obstacles, intelligent vehicles and mobile robots can improve the robustness of autonomous navigation functions, avoiding potential collisions. Additionally, in augmented reality systems, virtual objects can be placed in the real world based on depth maps estimated from a small camera sensor. While LiDAR and radar sensors can measure accurate distances, these sensors have intrinsic limitations including high costs and data sparsity. On the other hand, depth maps inferred from a depth-estimation model provide dense 3D information, consisting of pixel-level distances. Moreover, the depth estimates can be easily integrated with semantic information obtained from other image-processing modules without additional extrinsic calibration processes.

However, there are many challenges for estimating depth maps from fisheye camera images. The strong distortion of fisheye lenses and high-order distortion models directly affect the performance of depth-estimation models. While many deep learning methods have been proposed, fisheye camera models have not been thoroughly explored for depth-estimation algorithms so far. Moreover, the lack of fisheye camera datasets causes additional challenges for developing application-specific depth-estimation models. Existing fisheye camera datasets are mainly focused on object detection [1,2] and semantic segmentation tasks [3]. Regarding the application of autonomous parking systems, existing fisheye camera datasets only contain bird’s-eye view images [4,5], not providing the ground truth for the depth maps. To the best of our knowledge, there are two public datasets consisting of fisheye camera images for the depth estimation task. KITTI-360 [6] provides fisheye images and their corresponding LiDAR projections. However, this dataset only covers outdoor road environments and consists of non-overlapping 360° images, which were captured by two fisheye cameras. This non-overlapping setting leads to the limitations that the dataset cannot be utilized to train a depth-estimation model in a self-supervised manner based on a multi-view geometry. On the other hand, the WoodScape dataset [3] provides surrounding fisheye camera images, which were captured by four fisheye cameras. However, ground truth LiDAR projections of the WoodScape dataset have not been made publicly available yet, making further research difficult.

The main objective of this paper was to provide a novel fisheye camera dataset for developing depth-estimation algorithms and to conduct a comprehensive performance analysis of state-of-the-art depth-estimation models, which were developed for pinhole camera images. Our dataset holds significance as it is specialized for parking lot environments, different from existing datasets, which mainly focus on driving scenarios in road environments. Moreover, we propose a self-distillation technique for estimating depth maps from a fisheye camera. We demonstrate that the teacher–student learning framework is effective to improve the performance of depth-estimation networks, by integrating additional supervision from dense depth predictions. Our contributions can be summarized as follows:

- We propose a novel fisheye image dataset, which is specialized for depth estimation in underground parking lot environments.
- A comprehensive performance analysis was conducted for state-of-the-art models on both the public and real-world datasets.
- We demonstrate the effectiveness of the self-distillation technique for improving the depth estimation accuracy in the parking lot environment.

The remainder of this paper is organized as follows. Section 2 presents the related work, and Section 3 explains the public KITTI-360 dataset and our real-world dataset. Section 4 and Section 5 present the proposed method and the experimental results. Finally, Section 6 presents the conclusion.

## 2. Related Work

### 2.1. Monocular Depth Estimation

Convolutional neural networks (CNNs) have made great progress in the field of computer vision. The inherent properties of CNNs, including parameter sharing and local connectivity, have led to significant performance improvement in various tasks. Eigen et al. [7] proposed the first deep learning model for predicting depth maps, and this model is composed of CNN layers that integrate the coarse and fine information. Additionally, the scale-invariant loss proposed in [7] has been widely utilized for the training of depth-estimation networks. Subsequently, numerous CNN-based depth-estimation networks such as [8,9,10] have been proposed. These networks employ CNN-based encoder–decoder architectures to extract effective visual features. Bhat et al. [8] proposed LocalBins, which predicts the depth distributions of local neighborhoods at every pixel, passing decoder features through MLP layers. Lee et al. [9] and Song et al. [10] introduced the Local Planar Guidance layer and Laplacian pyramid technique in the decoder modules, respectively. Despite these studies, the limited receptive fields of CNNs and the loss of details during the downsampling process remain challenges for improving the accuracy.

Recently, transformer architectures [11] have been widely utilized in the depth estimation task. Transformer is an MLP-based architecture, which infers queries, keys, and values from tokenized patch regions, and it employs multi-head self-attention layers to integrate the global information. Dosovitskiy et al. [12] introduced a transformer model into image classification by implementing self-attention mechanisms within patch embeddings for the input images. The successful performance improvement of ViT [12] has led to the emergence of transformer-based networks in several vision tasks [13,14,15,16]. In depth estimation, Ranftl et al. [16] utilized ViT to estimate depth, yielding comparable performance without a CNN. Subsequently, significant advancements have been achieved in the transformer-based networks [17,18,19,20,21], which have exhibited superior capabilities in capturing comprehensive global information compared with previous CNN-based models. Kim et al. [21] proposed a hierarchical transformer encoder and a lightweight decoder that adaptively integrates local and global features. Moreover, there has been a trend of effectively integrating CNN and transformer modules, and these architectures are called hybrid models [22,23,24,25].

### 2.2. Distortion-Aware Deep Learning Models

The use of wide-angle lens cameras has many benefits in industrial perspectives due to the capability of capturing information from a wide FOV. For example, a single fisheye camera can cover more than 180° of the diagonal FOV, reducing blind spots. The growing interest has led to the adaptation of previous algorithms for pinhole camera images to wide FOV images. However, recognizing visual information from wide FOV images poses additional challenges due to strong distortion and insufficient public datasets. Moreover, most previous methods for wide FOV images were focused on object-detection and -segmentation tasks. In the object-detection task, deep learning methods have been proposed to address the problem of the distorted shapes of objects in wide FOV images [26,27], and several algorithms are specialized at detecting people in top-view images [28,29,30,31]. On the other hand, segmentation methods have been proposed for wide FOV images, by effectively handling the strong distortion characteristics [32,33,34,35,36].

Wide-angle lens cameras have been widely utilized in autonomous vehicles for reducing blind spots and enhancing safety. To obtain dense 3D information from a single camera, depth-estimation methods have been proposed that utilize wide FOV images. Kumar et al. [37] addressed the problem of different view-points between the LiDAR sensor and fisheye camera to utilize the sparse LiDAR projections as the ground truth data for the training of a depth-estimation network. In [38,39,40], geometric methods were employed in deep learning models to handle the strong distortion of wide FOV images. Recently, the WoodScape [3] dataset has been utilized for developing depth-estimation networks for fisheye camera images [41,42,43,44,45]

### 2.3. Knowledge Distillation

Knowledge distillation is a technique for training a student network that mimics the responses of a teacher network. The teacher–student learning pipeline was first employed by Hinton et al. for compressing neural network architectures [46]. To improve its effectiveness, various types of knowledge have been distilled into student networks such as attention maps [47] and relationships between samples [48]. Additionally, Lan et al. [49] adjusted the distillation weight for each instance to selectively transfer the teacher’s knowledge to students, and Guo et al. [50] proposed a knowledge-distillation method with high interpretability and performance by transmitting the class activation map. Several studies have demonstrated that distilling knowledge between neural networks with an identical architecture is beneficial to improve accuracy [51], and this is called self-distillation. For example, Lan et al. [52] proposed adaptive instance distillation, which attentively adjusts the weights of instance distillation loss, and it showed performance improvement for the self-distillation technique. Recently, the knowledge has been transferred between transformer architectures, to achieve better accuracy using smaller models [53].

The technique of knowledge distillation has been applied to monocular depth estimation to improve robustness and efficiency. Pilzer et al. [54] introduced knowledge distillation for self-supervised depth estimation, and depth estimates of a teacher network were distilled into a student network. Liu et al. [55] proposed two auxiliary loss functions for distilling both pairwise and holistic knowledge of a teacher network to improve the accuracy of a student network. On the other hand, Wang et al. [56] distilled the knowledge into a lightweight student network to infer depth maps on mobile devices, and Zhou et al. [57] proposed a self-distilled feature aggregation module to integrate low-scale and high-scale features, maintaining contextual consistency.

## 3. Datasets

### 3.1. KITTI-360

The KITTI-360 dataset [6] is a large-scale dataset that contains 320 k images and 100 k laser scans captured by driving in several suburbs of Karlsruhe, Germany. The KITTI-360 dataset was collected by equipping two 180° fisheye cameras on the left and right directions and a 90° perspective stereo camera at the front. A Velodyne HDL-64E and a SICK LMS 200 laser scanner were mounted on the robot to acquire the 3D information of the surrounding environments. This dataset comprises raw data, as well as semantic and instance labels in both 2D and 3D formats, enabling various applications such as 2D and 3D segmentation, 3D bounding box detection, novel view synthesis, and semantic SLAM. The KITTI-360 dataset also provides rectified images from the perspective stereo camera and fisheye images from the fisheye cameras. In our experiments, we used both the rectified and fisheye images to develop depth-estimation networks and to compare their performance. Point clouds acquired from the Velodyne HDL-64E were projected onto the 2D plane to obtain the ground truth depth maps. Both the RGB images and the depth maps have a resolution of 376×1408 px for the rectified images and 1400×1400 px for the fisheye images. Regarding the fisheye images, because the vertical FOV of the LiDAR sensor is 26.8°, the LiDAR projections provide valid distance information only within the region between 670 and 970 px in height.

### 3.2. JBNU-Depth360

Accurate depth estimation is important for autonomous vehicles in on-road driving and parking scenarios. Specifically, in parking lot environments, it is crucial to measure the distances to other vehicles and surrounding structures for recognizing unoccupied parking areas and avoiding potential collisions. However, existing datasets for depth estimation are mainly focused on outdoor driving scenarios. Therefore, it is not clear whether the models learned from these datasets perform well in parking lot environments. To address the limitations, we propose a novel fisheye image dataset acquired from underground parking lot environments.

**Figure 1:**
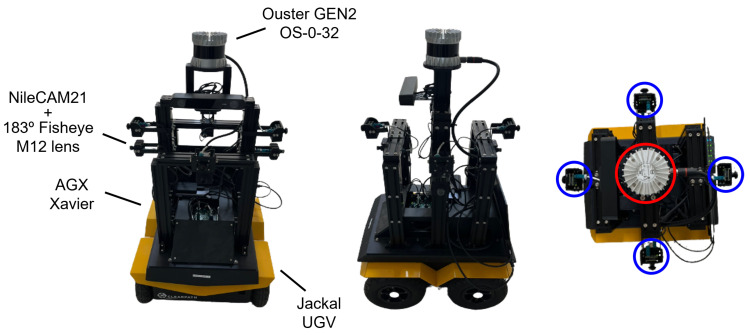
The sensor configuration (**left**,**middle**) and top-view image (**right**) of the mobile robot used for collecting the JBNU-Depth360 dataset. A Jackal UGV equipped with an AGX Xavier. An Ouster GEN2 OS-0-32 is located at the center of the robot to acquire the LiDAR projections of surrounding environments, and four NileCAM21 cameras with a 183° Fisheye M12 lens are equipped in the front, rear, left, and right directions. In the top-view image, LiDAR and camera positions are highlighted in red and blue, respectively.

Our dataset was collected for developing depth-estimation algorithms based on fisheye cameras, and it is referred to as the JBNU-Depth360 dataset. To acquire the dataset, we constructed a mobile robot equipped with an AGX Xavier on a Jackal UGV. Specifically, the mobile robot was equipped with four NileCAM21 cameras, each of which was assembled with a 183° Fisheye M12 lens. These four cameras were placed at the front, rear, left, and right directions, respectively. Additionally, an Ouster GEN2 OS-0-32 was located at the center of the robot to acquire the LiDAR projections of the surrounding environments. Because our mobile robot was designed for the acquisition of fisheye images and their corresponding LiDAR projections, we employed a LiDAR sensor with a vertical FOV of 90°. While KITTI-360 provides valid depth information only within parts of the fisheye images between 670 and 970 px in height, our LiDAR projections covered almost entire regions of the fisheye images. The sensor configuration and top-view image of the mobile robot are presented in Figure 1. Subsequently, a calibration process was conducted to compute the extrinsic parameters between the cameras and LiDAR. A checkerboard was utilized to calibrate the cameras and the LiDAR sensors, ensuring accurate spatial correspondence between the RGB images and the corresponding LiDAR projections.

**Figure 2:**
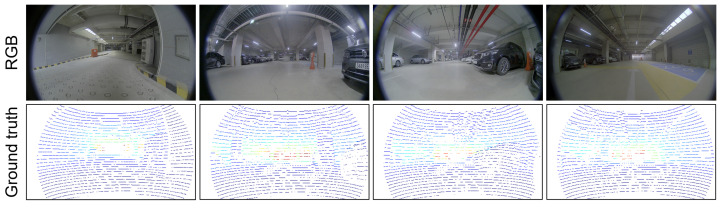
Examples of the JBNU-Depth360 dataset. RGB images and their corresponding ground truth data produced by LiDAR projections. Because the LiDAR projections are very sparse, 3×3 kernels are applied to the valid pixels for the visualization purpose. White pixels in the ground truth data indicate invalid LiDAR projections. Red and blue pixels indicate near and far distances, respectively.

The JBNU-Depth360 dataset was acquired from underground parking lot environments located at Jeonbuk National University, South Korea. The dataset consists of 6 sequences, and we sampled 4221 RGB images for the frontal camera by removing temporally adjacent images that contained similar visual information. LiDAR–camera calibration was conducted in each data-acquisition process to obtain the extrinsic parameters. Ground truth depth maps were produced by projecting 3D LiDAR points onto the image plane of the corresponding RGB images based on the extrinsic parameters. The RGB images and LiDAR projections were stored with a resolution of 1080×1920 px. After collecting the dataset, car plate numbers and faces were blurred in the RGB images to anonymize private information. Figure 2 presents examples of the RGB images and their corresponding LiDAR projections.

## 4. Knowledge Distillation for Monocular Depth Estimation

Ground truth data for supervised learning of depth-estimation networks are mainly based on depth maps, which are generated by projecting 3D LiDAR points onto the 2D image plane. These depth maps commonly have sparse depth information due to the intrinsic limitations of LiDAR sensors, which are unable to capture point clouds across the entire 3D space. Sparse depth maps contain insufficient information for understanding the comprehensive 3D structures of the surrounding environments, resulting in the suboptimal performance of depth-estimation networks. Especially when training with a dataset acquired from a single platform, we observed that depth-estimation networks easily infer unreliable depth information, producing striped depth patterns. To address this problem, we employed a knowledge-distillation technique that leverages both the sparse LiDAR ground truth and the dense depth map for supervision.

**Figure 3:**
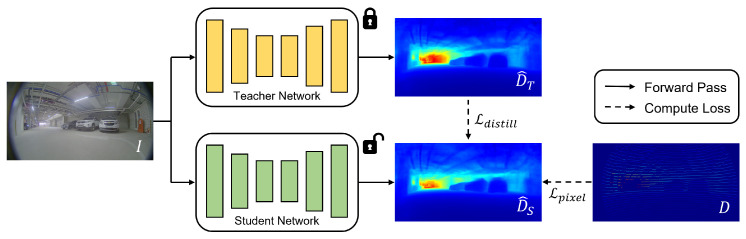
Overview of the knowledge-distillation process. The teacher network (yellow color) is trained by utilizing sparse LiDAR projections, and its trainable parameters are fixed during the knowledge-distillation process. After the training of the teacher network, the student network (green color) is trained by utilizing both LiDAR projections and estimated depth maps inferred by the teacher network. The process of knowledge distillation minimizes $L_{distill}$ and $L_{pixel}$, which are computed using ${\hat{D}}_{T}$ and *D*, respectively.

The process of knowledge distillation consisted of a teacher–student learning framework, as presented in Figure 3. Any deep learning model for monocular depth estimation can be utilized as the teacher and student networks, in the process of self-distillation. In the experiments, three existing models were employed to demonstrate the effectiveness of the self-distillation technique on the fisheye camera dataset. The teacher network was trained by utilizing sparse LiDAR projections, and its predicted depth maps were employed as dense supervision for the training of the student network. While the predicted depth maps were less accurate than the LiDAR projections, they provided depth information for entire pixels, allowing the training of a depth network with more-abundant information. During the training of the student network, the trainable parameters in the teacher network were fixed, and the gradients of the parameters in the student network were computed based on two functions consisting of pixel-level loss and distillation loss.

In Figure 3, an RGB image $I\in R^{H\times W\times C}$ is fed into the student network to infer the depth prediction ${\hat{D}}_{S}$. The pixel-level loss $L_{pixel}$ measures the distance between the predicted depth map ${\hat{D}}_{S}$ and the LiDAR ground truth *D*, and it is defined as follows.

(1) $$L_{pixel}=\alpha \sqrt{\frac{1}{n}\sum_{i}g_{i}^{2}-\frac{\lambda }{n^{2}}{\left(\sum_{i}g_{i}\right)}^{2}},$$

where $g_{i}$ is the logarithmic difference between ${\hat{D}}_{S}^{i}$ and $D^{i}$, which are the depth prediction and LiDAR projection at the *i*-th pixel, and it can be computed as $g_{i}=\log{\hat{D}}_{S}^{i}-\log D^{i}$. In (1), *n* denotes the number of pixels having valid ground truth values, and we set λ=0.85 and α=10 in the experiments. The pixel-level loss is based on the scale-invariant loss introduced by [7], and it enables the student network to learn the precise depth information measured by the LiDAR sensor.

To utilize the dense supervision produced by the teacher network, we defined a distillation loss based on the Structural Similarity Index (SSIM) loss [58]. The distillation loss is defined as (2), and it measures the distance between the predicted depth map ${\hat{D}}_{S}$ and the dense depth map ${\hat{D}}_{T}$.

(2) $$L_{distill}=\frac{1-SSIM({\hat{D}}_{S},{\hat{D}}_{T})}{2}.$$

This loss term encourages the student network to learn abundant depth information based on depth predictions. While computing the distillation loss, the window size of the SSIM was set to 7. The total loss function for the training of the student network is defined as a linear combination of the pixel-level loss and distillation loss functions as (3):

(3) $$L_{total}=L_{pixel}+\beta L_{distill}.$$

The weight parameter β was set to 0.1 in the experiments.

## 5. Experimental Results

Experiments were conducted to demonstrate the effectiveness of the proposed method for estimating depth maps from fisheye camera images in parking lot environments. In the experiments, the KITTI-360 and JBNU-Depth360 datasets were utilized to analyze the performance of the state-of-the-art depth-estimation models. KITTI-360 is a public dataset that provides both rectified and fisheye images, and experiments were conducted to compare the performance of previous models in both types of images. JBNU-Depth360 is our real-world fisheye camera dataset acquired from parking lot environments, and this dataset is available at https://github.com/EunjinSon1/JBNU-Depth360 (accessed on 15 December 2023). In the JBNU-Depth360 dataset, we conducted thorough experiments to analyze the effects of image pre-processing and the transfer learning of pre-trained parameters. Regarding the pre-processing, random cropping and image resizing were analyzed because these two approaches have been utilized to reduce memory consumption in previous literature without sufficient analysis. Moreover, we analyzed the effects of transfer learning in both quantitative and qualitative aspects. Finally, we demonstrated the effectiveness of the knowledge-distillation technique for improving the performance of the depth-estimation models.

### 5.1. Experimental Environments and Evaluation Metrics

Experiments were conducted in a hardware environment including an Nvidia GeForce RTX 3090 GPU and an AMD Ryzen9 5950X CPU. Python and Pytorch were utilized to implement the proposed algorithm. In the training process, BTS [9] and GLPDepth [21] were trained using the Adam optimizer [59] with a learning rate of $10^{-4}$. On the other hand, AdaBins [23] was trained using the AdamW optimizer [60] with a learning rate of $3.5^{-4}$. We trained the deep learning models for 20 epochs with a batch size of four in the experiments.

We evaluated the quantitative performance of the depth-estimation models based on the standard metrics used in the previous work [7]. The accuracy metric was measured by computing the ratio of pixels that had relative errors lower than a threshold value. The relative error at the *i*-th pixel was computed by $\delta =max(\frac{{\hat{D}}_{S}^{i}}{D^{i}},\frac{D^{i}}{{\hat{D}}_{S}^{i}})$, and we utilized three threshold values: 1.25, $1.25^{2}$, and $1.25^{3}$, following the previous work. We utilized six error metrics: absolute relative error (AbsRel), squared relative error (SqRel), root-men-squared error (RMSE), root-mean-squared logarithmic error (RMSE log), average log error (log 10), and scale-invariant logarithmic error (SILog). Among them, AbsRel and SILog were mainly analyzed in this Experimental Results Section, because AbsRel is an intuitive measure for understanding the degree of errors and SILog is the main metric for comparing previous algorithms in the original KITTI benchmark. Details about the accuracy and error metrics can be found in the previous work [7].

### 5.2. Experimental Results on the KITTI-360 Dataset

The KITTI-360 dataset consists of 9 sequences, and we split the dataset into 7 sequences for training and 2 sequences for testing. From the training sequences, 3000 images were randomly sampled from each sequence to construct a training dataset, and therefore, the training set consisted of 21,000 images. For constructing a test set, 1 frame was selected for every 10 frames within each test sequence, and the test set consisted of 974 images. In the case of rectified images, the training data were randomly cropped with a size of 352×704. On the other hand, fisheye images contained valid pixels within the region approximately ranging from 670 to 970 in the height direction and from 70 to 1370 in the width direction. Considering the characteristics of the fisheye images, we randomly cropped them with a size of 416×704 within the region ranging from 600 to 1016 in the height direction and from 0 to 1400 in the width direction.

Experiments were conducted on three existing models: BTS, AdaBins, and GLPDepth, and Table 1 presents the quantitative results for rectified and fisheye images in the KITTI-360 dataset. For the rectified images, GLPDepth outperformed BTS and AdaBins across all metrics, achieving 14.417 for the SILog metric. BTS showed comparable performance to GLPDepth in terms of AbsRel and log10. On the other hand, for the fisheye images, BTS resulted in superior performance to the GLPDepth and AdaBins models, achieving 0.122 for the AbsRel metric. We presumed that the pre-processing of random cropping critically affected the performance of the GLPDepth model on the fisheye images. The computational cost of our method is closely related to the model size, and the BTS, AdaBins, and GLPDepth models contain 48M, 78M, and 62M parameters, respectively. Overall, while fisheye images provide visual information in a wider field of view compared to rectified images, the quantitative performance of the depth-estimation models was degraded in the fisheye images. Our objective was to improve the performance of the depth-estimation models for fisheye camera images acquired from parking lot environments. We conducted more-thorough experiments on the JBNU-Depth360 dataset by analyzing the effects of pre-processing and transfer learning.

**Figure 4:**
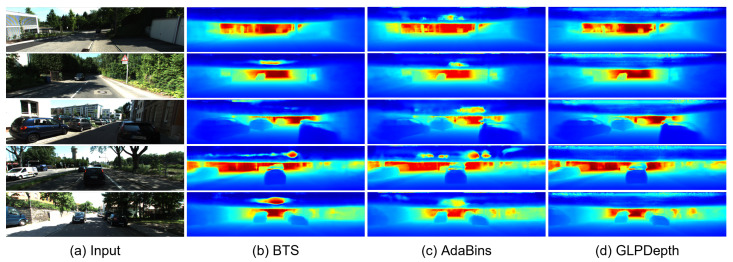
Qualitative results of depth-estimation networks for rectified images in the KITTI-360 dataset.

**Figure 5:**
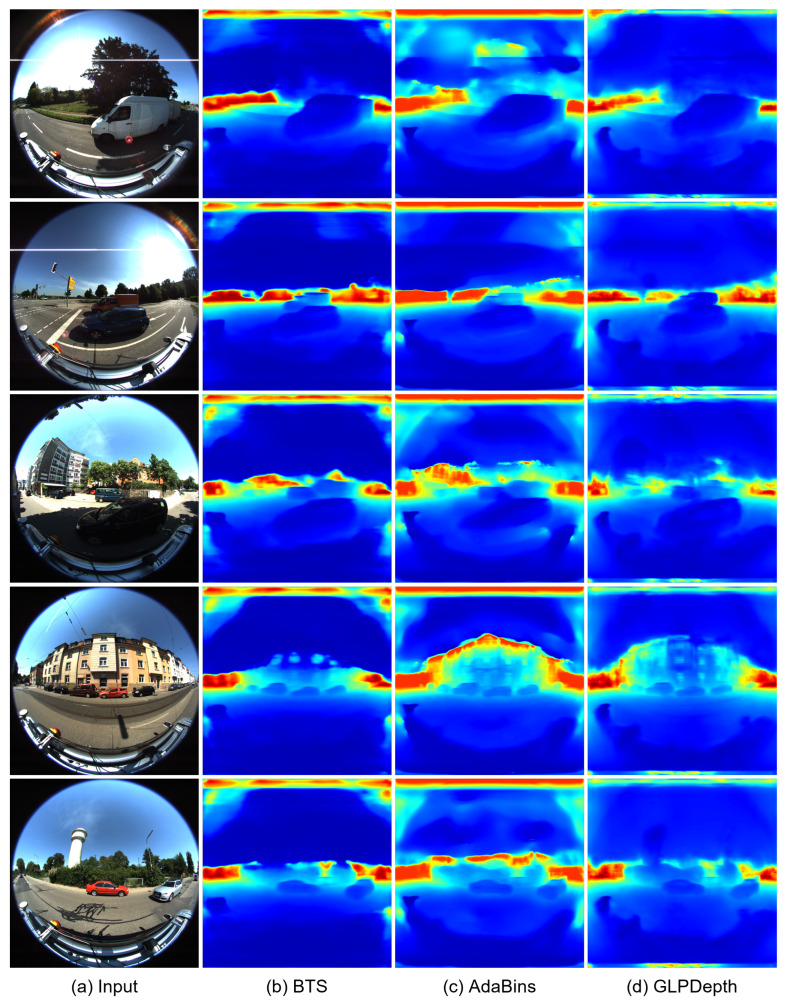
Qualitative results of depth-estimation networks for fisheye images in the KITTI-360 dataset. (**a**) indicates input images. (**b**–**d**) present depth maps inferred by BTS [9], AdaBins [23], and GLPDepth [21], respectively.

Figure 4 and Figure 5 present the qualitative results for the rectified and fisheye images in the KITTI-360 dataset, respectively. In Figure 4, the boundaries of vehicles and buildings are identifiable in the predicted depth maps. In the results for the rectified images, the BTS and GLPDepth models showed better quality for estimating distances for far regions, and this resulted in lower RMSE metrics compared to the AdaBins model in Table 1. On the other hand, regarding the fisheye images, all models inferred meaningful depth information only within the region with the height ranging from 670 to 970 px, as shown in Figure 5. The reason for the incorrect estimates outside of the region is because the ground truth of the KITTI-360 dataset only had valid distance information in the region of 670 and 970 px in height.

### 5.3. Experimental Results on the JBNU-Depth360 Dataset

The JBNU-Depth360 dataset consists of 6 sequences, and it was split into 5 training sequences and 1 test sequence. From the training and test sequences, we sampled 3114 images for the training set and 1101 images for the test set, by removing redundant images that contained similar visual information. In the following experiments, we present the performance of the depth-estimation models on the test set for analyzing the effects of pre-processing, transfer learning, and knowledge distillation.

Experimental results with the pre-processing of random cropping are presented in Table 2 and Table 3. This pre-processing has been widely utilized in previous work to reduce the memory consumption and training time. Table 2 presents the performance of the three deep learning models, which are initialized with the pre-trained parameters trained on the original KITTI dataset. The BTS model achieved 0.124 and 16.852 for the AbsRel and SILog metrics, outperforming the AdaBins and GLPDepth models. While the number of parameters in the AdaBins and GLPDepth models was 78 million and 62 million, which is larger than the BTS model consisting of 48 million parameters, the AdaBins and GLPDepth models showed unsatisfactory performance with the pre-processing of random cropping.

**Figure 6:**
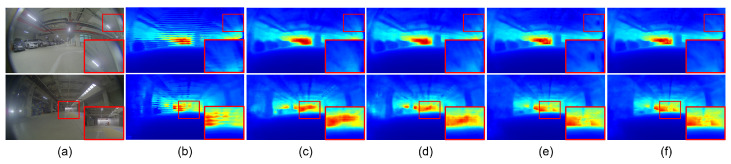
Qualitative results of BTS on the JBNU-Depth360 dataset. (**a**) indicates input images. (**b**) is the depth maps without TL and KD. (**c**) presents the results with a learning rate of $10^{-5}$. (**d**) is the depth maps with KD. (**e**) is the depth maps with TL, and (**f**) is those with TL and KD. Red boxes highlight the details of the predicted depth maps.

Table 3 and Figure 6 present the effects of transfer learning and knowledge distillation. When the BTS model was trained without using the pre-trained parameters, the quantitative performance was slightly improved from 0.124 to 0.120 for the AbsRel metric. However, striped noisy patterns were produced when the BTS model was trained without transfer learning, and it significantly degraded the quality of the predicted depth maps, as shown in Figure 6b,e. While striped noisy patterns were reduced with the use of a smaller learning rate, it affected the quantitative performance. By applying the knowledge-distillation technique, the SILog metric was improved from 15.782 to 15.623 and from 16.852 to 16.748 when the BTS model was trained with and without using the pre-trained parameters, respectively.

**Table 2:** Performance of BTS, AdaBins, and GLPDepth models on the JBNU-Depth360 dataset. The pre-processing of random cropping was applied to the input images. Accuracy and error metrics are denoted by ↑ and ↓, respectively.

Method	δ<1.25↑	$\delta <1.25^{2}↑$	$\delta <1.25^{3}↑$	AbsRel ↓	SqRel ↓	RMSE ↓	RMSE log↓	log10↓	SILog ↓
BTS [9]	0.834	0.975	0.994	0.124	0.198	1.315	0.177	0.053	16.852
AdaBins [23]	0.684	0.952	0.991	0.197	0.298	1.445	0.233	0.081	21.985
GLPDepth [21]	0.557	0.902	0.984	0.261	0.365	1.396	0.279	0.097	21.983

**Table 3:** The effectiveness of transfer learning and knowledge distillation on the JBNU-Depth360 dataset. Experiments were conducted using the BTS model based on the pre-processing of random cropping. TL denotes the use of transfer learning with the pre-trained weight for the original KITTI dataset. KD indicates the use of knowledge distillation. Accuracy and error metrics are denoted by ↑ and ↓, respectively.

TL	KD	δ<1.25↑	$\delta <1.25^{2}↑$	$\delta <1.25^{3}↑$	AbsRel ↓	SqRel ↓	RMSE ↓	RMSE log↓	log10↓	SILog ↓
		0.846	0.978	0.995	0.120	0.184	1.232	0.168	0.050	15.782
	✓	0.850	0.978	0.995	0.119	0.178	1.208	0.167	0.050	15.623
✓		0.834	0.975	0.994	0.124	0.198	1.315	0.177	0.053	16.852
✓	✓	0.836	0.975	0.994	0.124	0.198	1.298	0.175	0.052	16.748

**Figure 7:**
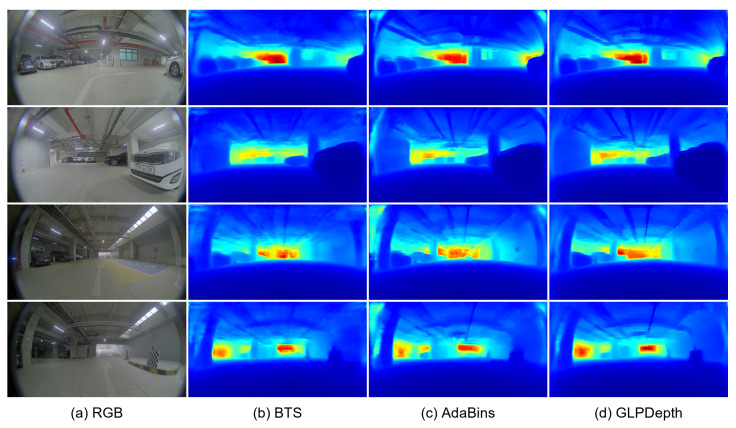
Qualitative results of depth-estimation networks on the JBNU-Depth360 dataset with the pre-processing of image resizing.

Experiments were conducted with the pre-processing of image resizing. We believe that the strong distortion of the fisheye camera images may deteriorate the performance of the depth-estimation models that were trained with the pre-processing of random cropping. Although the downsampling of the input images had an affect on precisely inferring the depth information at the object boundaries, we tried to improve the overall depth accuracy. In the experiments with the resizing pre-processing, the input images were downsampled into a size of the 512×960. Table 4 presents the quantitative results of the three depth-estimation models, with the pre-processing of image resizing and the transfer learning of the pre-trained parameters trained on the original KITTI dataset. With the resizing pre-processing, the GLPDepth model achieved 0.110 and 14.567 for the AbsRel and SILog metrics, outperforming the BTS and AdaBins models. By modifying the pre-processing method from random cropping to image resizing, the AbsRel metric of the GLPDepth model was improved from 0.261 to 0.110. Figure 7 presents the qualitative results of the depth-estimation models with the pre-processing of image resizing and transfer learning. The pixel-level distances to vehicles and free spaces are identifiable in the depth predictions inferred by the GLPDepth model.

**Figure 8:**
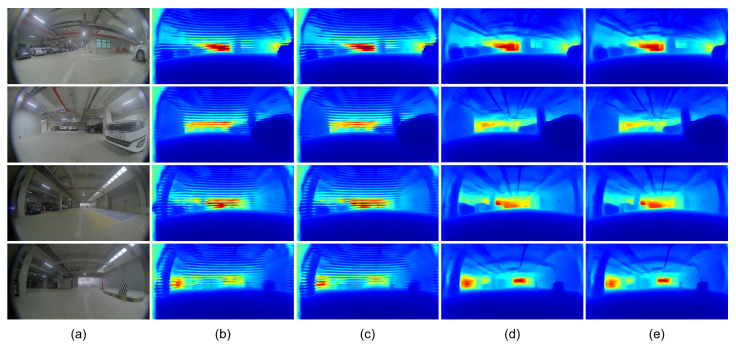
Qualitative results of GLPDepth on the JBNU-Depth360 dataset with the pre-processing of image resize. (**a**) indicates input images. (**b**) is the depth maps without TL and KD and (**c**) is with KD. Without TL, stripe noises were observed in the predicted depth maps. (**d**) is the depth maps with TL, and (**e**) is those with TL and KD.

Table 5 and Figure 8 present the effects of transfer learning and the knowledge-distillation technique with the pre-processing of image resizing. The experiments were conducted with the GLPDepth model, which shows the best performance in Table 4. Similar to the results in Table 4, the transfer learning of pre-trained parameters degraded the quantitative performance from 0.093 to 0.110 for the AbsRel metric and from 13.543 to 14.567 for the SILog metric. However, when the GLPDepth model was trained from scratch without transferring the pre-trained parameters, it resulted in striped noisy patterns, which deteriorated the quality of the predicted depth maps, as shown in Figure 8b. On the other hand, the knowledge-distillation technique consistently improved the SILog metric from 13.543 to 13.246 and from 14.567 to 14.340, when the GLPDepth model was trained with and without transfer learning, respectively. Figure 8 presents the qualitative results of the GLPDepth model with and without the use of transfer learning and the knowledge-distillation technique.

## 6. Conclusions

In this paper, we proposed a novel fisheye camera dataset called JBNU-Depth360 for the development of depth-estimation algorithms by using a knowledge-distillation technique. Existing fisheye camera datasets are mainly focused on outdoor driving scenarios and provide depth information only for a partial region of fisheye images. To address the limitations of existing datasets and expand the scope of depth-estimation methods, we established a fisheye camera dataset specialized for underground parking lot environments. The proposed dataset consisted of 4221 pairs of fisheye images and the corresponding wide FOV LiDAR projections, obtained from six driving sequences. To improve the performance of existing depth-estimation models, we introduced a self-distillation technique, which utilizes dense depth predictions as additional supervision. Experiments were comprehensively conducted on the KITTI-360 and JBNU-Depth360 datasets, using three existing depth-estimation models: BTS, AdaBins, and GLPDepth. The experimental results demonstrated that the self-distillation technique is effective at improving the performance of depth-estimation models, reducing the AbsRel and SILog error metrics by 1.81% and 1.55% on the JBNU-Depth360 dataset. We expect that our work will contribute to the advancement of research on depth-estimation algorithms for autonomous parking systems.

## Figures and Tables

**Table 1:** Performance of BTS, AdaBins, and GLPDepth models for rectified and fisheye images in the KITTI-360 dataset. Accuracy and error metrics are denoted by ↑ and ↓, respectively.

Image	Method	Evaluation Metrics								
		δ<1.25↑	$\delta <1.25^{2}↑$	$\delta <1.25^{3}↑$	AbsRel↓	SqRel↓	RMSE↓	RMSE log↓	log10↓	SILog↓
Rectified	BTS [9]	0.911	0.968	0.988	0.086	0.301	2.011	0.151	0.037	14.660
	AdaBins [23]	0.903	0.966	0.986	0.091	0.325	2.053	0.157	0.039	15.170
	GLPDepth [21]	0.914	0.971	0.989	0.086	0.291	1.936	0.149	0.037	14.417
Fisheye	BTS [9]	0.855	0.967	0.988	0.122	0.408	2.272	0.179	0.053	16.485
	AdaBins [23]	0.828	0.954	0.982	0.139	0.482	2.489	0.199	0.061	18.529
	GLPDepth [21]	0.719	0.921	0.970	0.175	0.648	3.047	0.249	0.081	22.997

**Table 4:** Performance of BTS, AdaBins, and GLPDepth models on the JBNU-Depth360 dataset. The pre-processing of resizing was applied to the input images. Accuracy and error metrics are denoted by ↑ and ↓, respectively.

Method	δ<1.25↑	$\delta <1.25^{2}↑$	$\delta <1.25^{3}↑$	AbsRel ↓	SqRel ↓	RMSE ↓	RMSE log↓	log10↓	SILog ↓
BTS [9]	0.834	0.971	0.995	0.124	0.212	1.316	0.175	0.051	16.210
AdaBins [23]	0.839	0.962	0.995	0.121	0.217	1.267	0.170	0.048	15.662
GLPDepth [21]	0.851	0.974	0.996	0.110	0.184	1.165	0.158	0.044	14.567

**Table 5:** The effectiveness of transfer learning and knowledge distillation on the JBNU-Depth360 dataset. Experiments were conducted using the GLPDepth model based on the pre-processing of resizing. TL denotes the use of transfer learning with the pre-trained weight for the original KITTI dataset. KD indicates the use of knowledge distillation. Accuracy and error metrics are denoted by ↑ and ↓, respectively.

TL	KD	δ<1.25↑	$\delta <1.25^{2}↑$	$\delta <1.25^{3}↑$	AbsRel ↓	SqRel ↓	RMSE ↓	RMSE log↓	log10↓	SILog ↓
		0.887	0.980	0.996	0.093	0.147	1.053	0.145	0.039	13.543
	✓	0.892	0.980	0.996	0.092	0.138	0.997	0.141	0.038	13.246
✓		0.851	0.974	0.996	0.110	0.184	1.165	0.158	0.044	14.567
✓	✓	0.860	0.979	0.995	0.108	0.171	1.150	0.156	0.044	14.340

## Data Availability

The KITTI-360 dataset is publicly available online. The public dataset can be found at https://www.cvlibs.net/datasets/KITTI-360, accessed on 22 October 2023. The JBNU-Depth360 dataset is available at https://github.com/EunjinSon1/JBNU-Depth360, accessed on 22 October 2023.
